# Athlete’s Personal Values and the Likelihood of Alcohol Use and Heavy Drinking during Adolescence

**DOI:** 10.3390/ejihpe14050080

**Published:** 2024-05-01

**Authors:** Juan Facundo Corti, Isabel Castillo, Agustin Miscusi, Vanina Schmidt

**Affiliations:** 1 Research Institute, Faculty of Psychology, University of Buenos Aires, Buenos Aires C1052AAA, Argentina; jfcorti@psi.uba.ar (J.F.C.); amiscusi@psi.uba.ar (A.M.); vschmidt@psi.uba.ar (V.S.); 2 Department of Social Psychology, Faculty of Psychology and Speech Therapy, University of Valencia, 46010 Valencia, Spain; 3 National Scientific and Technical Research Council (CONICET), Buenos Aires C1425FQB, Argentina; 4 Faculty of Psychology and Human Relations, Interamerican Open University, Buenos Aires C1147AAU, Argentina

**Keywords:** personal values, alcohol, binge drinking, adolescent, athletes

## Abstract

Sport is considered an exceptional activity for promoting healthy lifestyles, but the relationship between sport and alcohol consumption is inconclusive. Research on personal values may shed light on this issue and thus make it possible to find effective ways to prevent alcohol misuse in adolescents. The main objectives of this study were to explore the relationships between personal values and alcohol consumption amongst adolescent athletes and to validate the Portrait Values Questionnaire-21 (PVQ-21) in this population. A total of 914 athletes (aged 11–19; 55.4% female) participated in this study. Confirmatory multidimensional scaling analysis and confirmatory factor analysis were performed. Logistic regression models were fitted to assess the predictive power of personal values on alcohol use. Openness to change values positively predicted high-frequency alcohol use and high-frequency heavy episodic drinking, whereas the opposite occured with athletes who held conservation values. Furthermore, the probability of presenting heavy episodic drinking was negatively associated with conservation values. Finally, the PVQ-21 presented adequate psychometric properties to assess personal values among adolescent athletes. It is crucial to consider the personal values of adolescent athletes when promoting healthy lifestyles through sport.

## 1. Introduction

Alcohol consumption is one of the main factors of morbidity and mortality among adolescents [1]. This behavior itself is a threat to public health, but it also clusters with other risk behaviors and persists into adulthood [2]. The Region of Americas have some of the highest prevalence rates of alcohol consumption in the world, and in terms of drinking patterns, 18.3% of adolescents aged 15–19 presented heavy episodic drinking (HED) in the past year, which rises to 22.6% in South American countries [1]. HED is defined as the ingestion of a large amount of alcohol (approximately more than 60 g of pure alcohol) in a short period of time (3 to 4 h), and it is the drinking pattern with the greatest impact on adolescent health [3]. This situation becomes an obstacle not only to achieving the third Sustainable Development Goal (good health and well-being), but also has an impact on the other goals and targets of the 2030 Agenda [1]. Hence, it is of great importance to identify strategies to prevent risky alcohol use in adolescence.

Sport is considered a privileged activity for promoting healthy lifestyles [4], but the literature regarding its relationship with alcohol consumption is inconclusive [5,6]. For this reason, current studies tend to incorporate aspects of the activity and the athletes that may better explain the positive health effects of sport participation. For example, some studies suggest that the enjoyment, engagement, and personal fulfilment within the sport activity are associated with a lower frequency of alcohol consumption in adolescents [7,8,9]. Likewise, the individual characteristics of athletes affect the perception they have of their practice and may increase or decrease their psychological well-being [10] and consequently the likelihood of adopting risk behaviors during adolescence [11]. Among the dispositional variables that may have a role in the association between sport participation and alcohol consumption, the athletes’ personal values could have explanatory potential, as values influence how people perceive, feel, and behave [12]. Consideration of the individual characteristics of adolescent athletes, such as their personal values, may be the key to enhancing positive youth development through sport [13,14], thus preventing the use of alcohol at an early age. A major reason for studying values is based on the hypothesis that they have a significant impact on behavior and have explanatory and predictive potential [12]. For example, the literature suggests that values have consistent association with a variety of behaviors, such as consumer choices [15]. However, with some exceptions, little attention has been paid to the relationship that can be established between personal values and alcohol consumption behavior. Some of these studies are discussed below.

**Figure 1:**
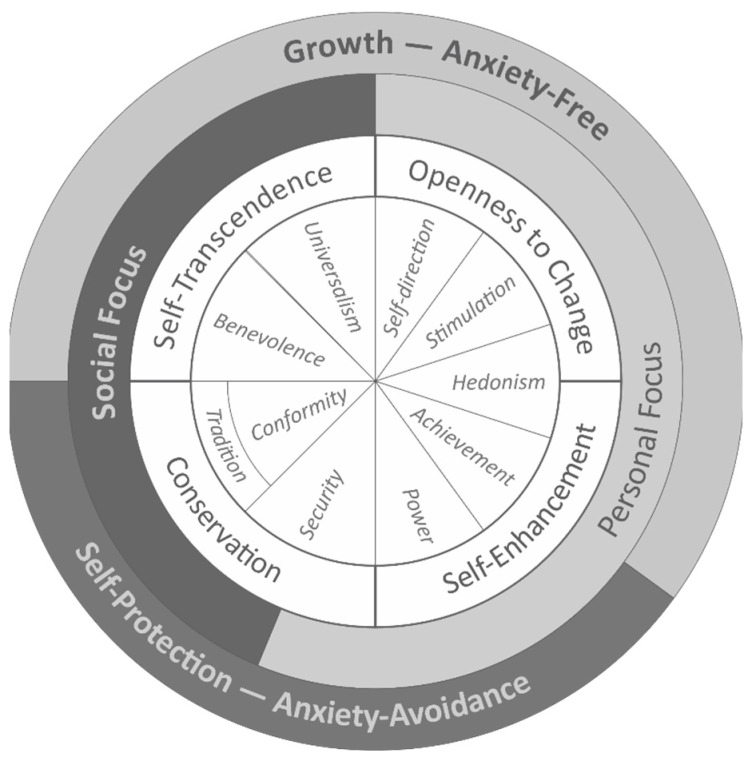
Theoretical model of relations among ten motivational types of values [17].

Schwartz’s theory of personal values [16,17] is a solid framework to understand values in sport and non-sport contexts [12,13,18]. Personal values are defined as desirable goals, stable across situations and time, that serve as guides for the conscious organization of emotions, attitudes, and behaviors [17]. Ten value domains are included in Schwartz’s original theory, with a circular structure where values that are close in structure are values that are compatible with each other (e.g., achievement and power), while opposite values in structure are values that would conflict with each other (e.g., power and universalism). This structure is organized into four higher-order motivations that form two bipolar and opposite dimensions (see Figure 1). One dimension opposes values that emphasize independence of judgment and action and favor change (openness to change) with those that emphasize preservation of traditions and protection of stability (conservation). The second dimension involves self-enhancement versus self-transcendence, contrasting values that emphasize the pursuit of personal success and dominance over others with those values that emphasize acceptance of others as equals and concern for their well-being [17]. Further classification of values distinguishes them according to their focus; self-transcendence and conservation are values with a social focus, whilst self-enhancement and openness to change are values with an individual focus. Finally, this theory of personal values also distinguishes between those values that promote anxiety-free growth (i.e., conservation and self-enhancement) and those values that prioritize self-protection or anxiety avoidance (i.e., self-transcendence and openness to change, see Figure 1). Although the original theoretical model was later refined by subdividing the original ten values into 19 basic values, both theoretical frameworks (classic and refined) are considered valid in the prediction of positive and negative outcomes derived from these values [17]. This universal circular structure facilitates a methodical association between values and behaviors [19].

Sports have been considered a good scenario to transmit personal values [13]. The scientific literature suggests that self-transcendence values are related to different desirable outcomes in sports, such as coaches’ transformational behavior [18], athletes’ intrinsic motivation [20], prosocial attitudes [13], and their personal and social skills [14]. On the contrary, self-enhancement values are positively associated with extrinsic motivation and amotivation in sports [20], antisocial attitudes [13], and antisocial behavior [21].

Studies that specifically target alcohol consumption found that values of openness to change and self-enhancement favor alcohol use, while conservation and self-transcendence values are associated with lower consumption [22,23]. In terms of the basic values, achievement and hedonism are associated with higher consumption and universalism, tradition, and conformity with lower alcohol use [24]. Specifically, during adolescence, having self-transcendence values were found to have a negative association with alcohol use, while openness to change values were positively associated with substance use problems [25]. As far as we know, no study has examined the associations between alcohol consumption and personal values among adolescent athletes. However, the present study aims to address this gap.

With respect to sex differences in alcohol use, numerous studies have identified that male adolescents tend to consume alcohol more frequently and in larger amounts [1]. However, in the last decade, Argentina has seen an upward trend in alcohol consumption among females, catching up with males [26]. Moreover, the recent Global School-based Student Health Survey found that young females in Argentina exhibit higher rates of HED, more drunkenness events, and more frequency of alcohol consumption (in the past month) compared to their male peers [27]. When considering age differences, there is a greater consensus that older adolescents consume more alcohol and more frequently [28,29,30].

Based on Schwartz’s theoretical framework, several instruments have been developed to assess personal values. Among these, the Portrait Values Questionnaire has three versions that are suitable for the assessment of adolescence values: the PVQ-21, PVQ-40, and PVQ-R, which consist of 21, 40, and 57 items respectively [31]. Although some validation studies were conducted in Argentina, where this study is being carried out, they have mainly been aimed at the adult population [32,33]. Fernandez Liporace et al. [34] conducted the only study on the assessment of personal values in adolescents using the PVQ-40. Beramendi and Zubieta [32] conducted a comparative analysis of the PVQ-21 and the PVQ-40 in a sample of adults and determined that the former presented better fit indices in both multidimensional scaling analysis (MDS) and confirmatory factor analysis (CFA). It is noteworthy that the latter study was the only one of the above-mentioned validations to effectively reproduce the value circle structure using MDS and CFA. No validation studies of the PVQ-21 in adolescent athletes were found. Furthermore, the PVQ-21 has not been found in Latin-American adolescents. Nevertheless, the number of items is significant for the assessment of adolescents, so it is crucial to validate the version with fewer items (i.e., PVQ-21) in this population.

Based on the given information, this study aims to achieve two main objectives. First, it aims to explore the relationships suggested in the literature between personal values and alcohol consumption in adolescent athletes while controlling for sex and age. For this purpose, we hypothesize that (1) social focus values (i.e., conservation and self-transcendence) have a negative correlation with alcohol use, whilst (2) personal focus values (i.e., openness to change and self-enhancement) are positively associated with alcohol consumption. It is expected that these relationships will become stronger as the level of sport practice increases, and therefore, we also hypothesize that (3) seasons of sport practice, weekly frequency, and duration of training sessions will moderate the aforementioned associations. Additionally, we expect (4) age to be associated with greater alcohol use whilst (5) sex is not expected to have a significant correlation. An exploratory analysis will also be conducted to evaluate the predictive capacity of the ten basic values on alcohol use. Secondly, this study aims to validate the PVQ-21 with a sample of adolescent athletes, testing the circular structure of its items and thus providing evidence of validity using MDS and CFA, respectively.

## 2. Materials and Methods

### 2.1. Participants

Participants were 914 adolescents (44.6% male, 55.4% female) aged between 11 and 19 years (M = 15.57, SD = 1.64; see Table 1 for the age distribution), who were members of sports teams competing at the regional level (basketball, handball, hockey grass, rugby, soccer, and volleyball) from Buenos Aires, Argentina. Most of the athletes (78%) had participated in their sport for four or more seasons, and 5.4% had participated in the national team at least once. On average, the teams practiced three times per week (M = 3.08; SD = 0.92), and all played at least one game on weekends.

### 2.2. Instruments

Personal values were measured using the Argentinean version [32] of the 21-item Portrait Values Questionnaire (PVQ-21) [35], by asking players to indicate how similar they are to gender-matched individuals who are described in terms of their important values. The questionnaire starts with the item, “How much is this person like you?”, and responses ranged from 1 (not like me at all) to 6 (very much like me). Items contain two statements describing a person: one statement expresses how important a particular value is for a person (e.g., “It’s very important to him to help the people around him”) and the other one describes the person’s desire for that value (e.g., “He wants to care for their well-being”). Every basic value of the ten proposed by the model is measured by two items, except for universalism (3 items). The four higher-order dimensions (i.e., self-transcendence, self-enhancement, openness to change, and conservation) are obtained by averaging between 4 and 6 items of the questionnaire. The reliability of this instrument has already been demonstrated to be good (α > 0.68) [36]. Cronbach’s alphas in this study were also acceptable (α from 0.63 to 0.76).

Adolescents’ alcohol consumption was assessed with the Argentinean adaptation [37] of the short version of the Alcohol Use Disorder Identification Test (AUDIT-C) [38], administered under written questionnaire. Through three items with a five-point Likert scale, where lower scores indicate lower alcohol use, this test measures frequency of drinking and presence and frequency of HED. The three AUDIT items assess the general trend of these drinking behaviors without delimiting a specific time window, as do other items in the test. To measure HED presence, the subject is asked to indicate the number of standard drinks consumed in a short period of time (more than 4/5 SD = 60 Gr/cc pure alcohol for women and men respectively, in approximately three or four hours). The AUDIT-C showed high sensitivity and low specificity, differences in the optimal cut-off point according to gender, acceptable internal consistency, and high test–retest stability in the Argentine population [39].

### 2.3. Procedure

A convenience sample of 18 team sports clubs was contacted, and the researchers explained the characteristics of this study, requesting collaboration. After institutional approval, participants were informed about the procedure and then completed the questionnaires voluntarily and anonymously during a 20 min period after practice. No specific definitions of the constructs measured were given to participants prior to their response; only that their honest answers would help in understanding how sport participation could relate to healthier lifestyles was stated.

### 2.4. Data Analysis

To evaluate whether the PVQ items replicated the theory-based structure of personal values, an ordinal confirmatory multidimensional scaling analysis was performed. This technique estimates the distances between items according to the correlation matrix and then plots these distances to reveal the structure of the data [40]. Specifically, the confirmatory approach of this analysis seeks to find an optimal theory-consistent solution with an external initial configuration of items and the distances between them. The unit circle was used as a reference to trigonometrically determine the coordinates of the initial configuration. The raw values were used to perform this analysis. A Stress-1 index of 0.05 or less indicates a good MDS solution, while less than 0.20 is considered acceptable [41]. A permutation test [42] with 100 replications was conducted to evaluate the significance of the Stress-1 index.

As recommended by Cieciuch and Schwartz [43], the magnifying glass strategy was used when performing the CFA to assess the internal structure of the PVQ-21. This approach consists of creating four different CFA models, one for each higher-order value (self-transcendence, conservation, self-enhancement, and openness to change). Unweighted least squares were used, which is a satisfactory estimator given the ordinal nature of the data and the sample size [44]. Four fit indexes were used to assess the goodness of the fit of the models: RMSEA, PCLOSE, CFI, and SRMR. With respect to cut-off points, RMSEA values < 0.05, PCLOSE > 0.05, CFI > 0.95, and SRMR < 0.08 indicate a good model fit [45]. According to the MDS solution in this study, hedonism was considered as part of openness to change.

Descriptive analyses of the consumption variables were carried out. Logistic regression models were fitted to assess the predictive power of personal values on alcohol use. For this purpose, the three consumption variables were dichotomized. Following Schmidt et al. [9], for frequency of consumption (FC), a low-frequency group was formed by subjects who drink once a month or less and a high-frequency group by subjects with regular or frequent consumption (twice a month or more). For the presence of HED, groups were formed for absence of HED (less than 4 SDs for females and less than 5 SDs for males) and presence of HED (4 or 5 SDs or more for females and males, respectively). For the variable frequency of HED, groups of low frequency of HED were formed by subjects who reported binge drinking once a month or less, and groups of high frequency of HED were formed by those who reported binge drinking once a week or more.

Simple logistic models with sex and age as predictors were fitted for each alcohol consumption variable. Models were then fitted with each higher-order value, controlling for sex and age. This study also examined the moderating effects of seasons of sport practice, weekly frequency, and duration of training sessions. A final model was then estimated for each consumption variable with the control variables and the personal values that were significant. To test the prediction accuracy, the cross-validation technique was implemented, selecting a random subset of 70% of the cases to fit the models and 30% to test their accuracy. The cut-off points for classifying the subjects were selected by considering the probability of maximizing both the sensibility and specificity of the models’ predictions. Finally, in order to assess which aspects of the higher-order values were more strongly associated with alcohol consumption, models were fitted using the basic values as predictors. Given the multicollinearity evidenced in the psychometric phase and in previous studies [32], models were fitted with each one of the ten values separately and with sex and age as control variables in all models. All analyses were performed using R 4.1.2 [46].

## 3. Results

**Figure 2:**
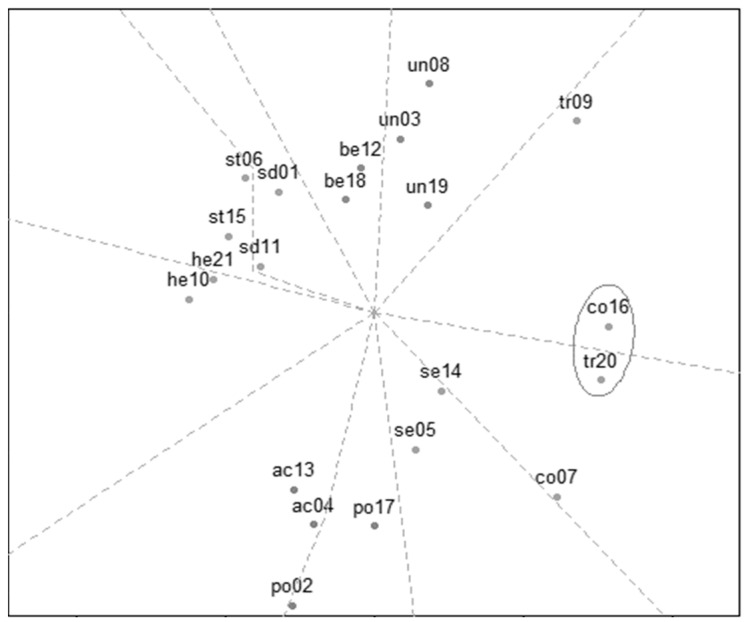
Multidimensional scaling (MDS) analysis of the 21-item Portrait Values Questionnaire (PVQ-21). *Note*. ac = achievement, be = benevolence, co = conformity, he = hedonism, po = power, sd = self-direction, se = security, st = stimulation, tr = tradition, un = universalism; the sections delineated by the dotted lines represent the area occupied by each basic value. The circle indicates the items located outside their respective sections.

The two-dimensional projection of the MDS solution is shown in Figure 2. An acceptable Stress-1 index of 0.14 was obtained and this indicator was significantly lower than the one obtained with permuted data (*p* < 0.001).

The spatial distribution of the solution found shows deviations from the theoretical circular motivational model originally proposed. Specifically, item 16 (conformity) and 20 (tradition) are inverted and placed equidistant from the center and not in the same sector as theorized. Furthermore, benevolence and universalism are also misplaced. The former is on the openness to change side and the latter on the conservation side. Despite this misplacing, the overall circular structure is replicated and the higher-order values have an accurate location in the MDS analysis.

Table 2 shows the global fit indexes of the CFA carried out. Although not all indexes were within the expected range, all four models showed good overall fits. Additionally, some values presented strong covariances. The associations power–achievement (self-enhancement), self-direction–stimulation (openness to change), security–tradition (conservation), and conformity–tradition (conservation) presented standardized factor loadings above 0.85, indicating multicollinearity.

No sex differences were found in alcohol consumption variables. The descriptive analysis of these variables is shown in Table 3.

Simple logistic regression models were estimated with sex and age as predictors for each alcohol consumption variable. The age models were significant for all three criterion variables (frequency of consumption: χ^2^ (1) = 33.83, OR = 2.02, CI_95_ = [1.57; 2.68]; presence of HED: χ^2^ (1) = 74.51, OR = 2.06, CI_95_ = [1.72; 2.5]; frequency of HED: χ^2^ (1) = 50.19, OR = 2.46, CI_95_ = [1.86; 3.36]). The sex models were not significant for any of the consumption variables.

When the effect of each value was evaluated separately, the self-transcendence and self-enhancement models were not significant in any of the tested models. The probability of having a high frequency of consumption or high frequency of HED was significantly predicted by conservation and openness to change. The probability of HED presence was only predicted by values of conservation. None of the moderation effects (i.e., seasons of sport practice, weekly frequency, and duration of training sessions) were statistically significant. The final multiple regression models are presented in Table 4.

Table 5 presents the results for each basic value model. None of the self-transcendence (i.e., universalism and benevolence) or self-enhancement (i.e., achievement and power) basic values were significant predictors of the consumption variables. When considering the frequency of consumption, all openness to change (i.e., self-direction, stimulation, and hedonism) and conservation (i.e., security, conformity, and tradition) basic values were significant, except for tradition. Similarly, tradition was the only non-significant conservation basic value when predicting the presence of HED. Finally, only hedonism (openness to change) was a significant predictor of the frequency of HED.

## 4. Discussion

The main goals of this study were twofold: first, to analyze the relationship between personal values and alcohol consumption behaviors in adolescent athletes, in order to protect this population from high-frequency and high-risk patterns of alcohol use; secondly, to examine the circular structure and construct validity of the 21-item Portrait Value Questionnaire (PVQ-21) in the sport setting, which will strengthen the findings of the first aim.

Starting with the second objective, the PVQ-21’s overall structure, as revealed by the multidimensional scaling analysis (MDS), reflects the theoretical distribution. The personal values are arranged in a circular disposition, with the four opposite dimensions on two axes: self-transcendence versus self-enhancement and openness to change versus conservation. The minor misplacements observed in this study were also present in previous research [32]. The repositioning of benevolence and universalism could be interpreted as a result of the shared motivations with the new adjacent values [32]. Adolescent athletes understand that ensuring the welfare of others outside their immediate environment (i.e., universalism) is connected to the respect for cultural values (i.e., tradition) and adherence to social norms and expectations (i.e., conformity). In Argentina, many adolescents, especially those who attend night-time events, engage in or witness fights with other adolescents, so it is not unreasonable for them to see universalism as synonymous with controlling impulses that could cause harm to others. Conversely, adolescent athletes perceive that enhancing the well-being of those closest to them (i.e., benevolence) is integral to pursuing autonomy and independence (i.e., self-direction). In this vital stage of identity formation, the peer group is of great relevance. Adolescent athletes probably perceive that the search for their own autonomous and independent identity is accompanied by the care and concern for their equals. In terms of the proximity of conformity and tradition to the center, rather than the latter being on the periphery of the former, athletes do not appear to differentiate between the level of abstraction of the objects they are subject to, which could distinguish one value from the other [47].

The confirmatory factor analysis models yielded goodness-of-fit indicators similar to those in previous literature [32], supporting the hypothesized structure. Additionally, multicollinearity issues detected in this study with athletes are also present within the general population [32]. In conclusion, the PVQ-21 exhibits adequate psychometric properties among adolescent athletes and provides evidence supporting the theoretical typology of the ten values and the assumed four second-order factors.

Similar to the results found in previous research conducted with Argentine and Spanish adolescents [48,49], the prevalence of risky alcohol consumption in this study was low. However, previous studies with adolescent athletes have shown high-frequency and high-risk patterns of alcohol use [7]. These findings indicate that sport participation alone does not lead to reduced alcohol consumption, so it is important to examine factors surrounding sport participation to understand its impact on alcohol use.

Our first and second hypothesis stated that social-focused values (i.e., conservation and self-transcendence) would have a negative correlation with alcohol use, while personal-focused values (i.e., openness to change and self-enhancement) would be positively associated with this outcome. When examining drinking frequency, the two hypotheses were partially confirmed. High drinking frequency was less likely when conservation values were held, while it became more likely when openness to change values were held. Previous research has found similar results, both in basic values [25] and at the level of higher-order dimensions [22]. This makes sense given the emphasis on social values (focusing on the welfare of others) endemic to conservation values and the emphasis on personal values (focusing on one’s own well-being) inherent to openness to change values. However, neither self-transcendence values nor self-enhancement values significantly predicted the likelihood of high-frequency drinking.

Regarding basic values, adolescents seeking pleasure and sensory gratification (hedonism), novelty and excitement (stimulation), and independence and exploration (self-direction) are more likely to have multiple drinking events per month (i.e., high-frequency alcohol use). Complementarily, seeking security and stability in relationships and in themselves (security) and avoiding actions that may upset or harm others or violate social norms (conformity) are associated with a lower frequency of alcohol consumption.

When examining the presence of heavy episodic drinking (HED), the only significant correlation was with the conservation value: for every unit increase in conservation value, the likelihood of engaging in this HED pattern decreased by 30.2%. Specifically, our findings suggest that athletes who prioritize security and conformity values are more inclined to abstain from alcohol consumption. Similar results were found in the general population, where tradition was associated with a reduction in alcohol consumption rather than security value [24]. Nonetheless, the measuring of alcohol consumption in their study integrated frequency and quantity of alcohol consumption per occasion, and it is important to consider that significant cultural differences, such as those between Norwegian and Argentinean societies, may lead adolescents to have different interpretations of what tradition means. Adolescents who prioritize a high value on security in their immediate environment or inhibiting actions that may be upsetting or harming to others are less likely to develop HED.

Similar findings emerged for HED frequency as for consumption frequency, with both conservation and openness to change values being significant predictors while self-transcendence and self-enhancement were not. Only high hedonism among the basic values increased the risk of high HED frequency. Interestingly, none of the models including basic conservation values separately were significant. It seems that the lower frequency of HED is not related to any one particular aspect (i.e., basic values) of the conservation values, but rather to the combination of its three components.

Self-transcendence and self-enhancement values were not found to be predictive of any of the alcohol use variables examined in this study, including the frequency of drinking episodes, presence of HED, and frequency of HED. Although some studies have found moderate relationships between these variables in the general population [24,50] and specifically in adolescents [25], openness to change and conservation are the only values that can be conceptually considered strong predictors of alcohol consumption. This is due to their strong motivation towards or against novel sensation seeking and sense stimulation. According to Schwartz’s theory [31], the axis that opposes openness to change and conservation synthesizes a basic universal tension in every individual between being independent, challenging, and changing, on one hand, and self-restriction, preservation of the past, order, and resistance to change, on the other. Hence, adolescent athletes who prioritize conservation values over openness to change are less likely to participate in high-risk behaviors, such as alcohol consumption and binge drinking.

It is noteworthy that hedonism is the openness to change value most closely associated with alcohol consumption. One of the reasons for consuming is the pursuit of pleasure and social interaction, which provides its own positive reinforcement [51]. In a recent local survey, 72.9% of participants refer to “pleasure, curiosity and search for new experiences” as principal reasons for their drinking behavior [30]. Hence, individuals who exhibit higher levels of hedonism tend to seek alcohol for its pleasurable effects. This aligns with the advertising strategies employed by the alcohol industry, which emphasize the rewards and enjoyment of consumption [24]. However, the lack of a relationship between stimulation and HED is remarkable. It is plausible that adolescents who prioritize stimulation often consume alcohol, but not necessarily in a risky way, unlike “hedonists” who frequently engage in high-frequency harmful alcohol consumption for pleasure.

The moderating effects of seasons of sport practice, frequency, and duration of training sessions (hypothesis 3) were not significant, thus these relationships between personal values and alcohol consumption are also present beyond the sporting level. Previous studies suggest that it is not sport participation per se that is related to alcohol consumption and well-being of young people, but the quality of the activity experiences [52,53]. In this sense, our results go one step further and these variables not only did not predict alcohol consumption, but also did not affect how personal values were associated with this outcome.

The fourth hypothesis of the study established that the age of the participants would be a significant predictor for all three consumption variables. This hypothesis was confirmed, leading to the conclusion that the risk of having high-frequency alcohol consumption, presence of HED, and high-frequency HED increases twofold with each passing year of age (increases of 102.2%, 106.2%, and 145.5%, respectively). While Márquez et al. [54] reported comparable findings regarding the higher prevalence of drinking alcohol among adolescents aged 16–17 compared to those aged 14–15, the effect size found in the present study was larger.

Although in many countries, young males consume more than females, in Argentina women have similar levels of alcohol consumption to men [26]. Consequently, our final hypothesis was that there would be no differences between the sexes. Nevertheless, the present study found that the risk of presenting HED among males was 52% lower than among females. Unlike what happened in the past, where being male was strongly linked to alcohol consumption (both in terms of frequency and intensity), the relationship between sexes and alcohol use seems to be less clear today. This study found that women are at higher risk of HED, which may reflect changes in social norms that indicate that alcohol consumption is no longer restricted to a single sex.

There are some limitations to this study. It is a cross-sectional study, which does not allow to establish causal relationships between the variables analyzed. The predictive capacity of some of the models presented is suboptimal, possibly due to the complexity of the behavior under study, which involves a multiplicity of factors not included (e.g., sociodemographic, family, personality, etc.). Additionally, generalizability may be enhanced by using samples beyond the sporting population, although the sample was deliberately chosen with the aim of analyzing the role of personal values on alcohol consumption in the sporting population. Furthermore, it is recommended to incorporate qualitative research methods, such as conducting focus groups and interviews with adolescent participants, to obtain more in-depth knowledge about how their values impact their alcohol consumption behaviors. Finally, future research could expand the sample size and age range to explore possible changes in how values affect drinking at different stages of life.

Despite these limitations, the data provided in this study contribute to the literature by providing information about the link between values and alcohol consumption behaviors in a sample of adolescent athletes. This extends previous research and suggests that values play an important role in explaining the assumption of risk behaviors.

## 5. Conclusions

The findings of this study contribute to the existing understanding of the importance of values in alcohol consumption among adolescent athletes. As guides of action in people’s life, we hypothesized that values would be potential predictors of alcohol use in the specific domain of sport.

In general, the present study revealed conceptually coherent relationships between particular basic values and values of dimensions and frequency of drinking. Athletes who hold openness to change values are more likely to drink alcohol frequently and to have episodes of heavy drinking more frequently, whereas the opposite occurs with athletes who hold conservation values. In addition, conservation emerged as a negative predictor of the presence of heavy episodic drinking. Finally, the PVQ-21 constitutes a valid instrument for the assessment of personal values in this population.

## Figures and Tables

**Table 1:** Age distribution.

Age	*n*	%	Cumulative Frequency	Cumulative Percentage
11	3	0.33	3	0.33
12	43	4.70	46	5.03
13	120	13.13	166	18.16
14	195	21.34	361	39.50
15	171	18.71	532	58.21
16	179	19.58	711	77.79
17	136	14.88	847	92.67
18	61	6.67	908	99.34
19	6	0.66	914	100

**Table 2:** Confirmatory factor analysis: goodness of fit indexes for each higher-order value.

Models	*χ*^2^/*df*	CFI	RMSEA	SRMR	PCLOSE	Factor Loadings
Self-transcendence	0.52	1.00	0.00	0.12	0.99	0.58–0.73
Conservation	12.94	0.98	0.11	0.03	<0.01	0.31–0.64
Self-enhancement	1.38	1.00	0.02	0.01	0.64	0.57–0.80
Openness to change	6.92	0.99	0.08	0.05	0.01	0.57–0.77

*Note.* CFI = comparative fit index, *df* = degrees of freedom, PCLOSE = probability of close fit, RMSEA = root mean square error of approximation, SRMR = standardized root mean square residual.

**Table 3:** Descriptive analysis of alcohol consumption variables.

Variable		n (%)			χ^2^ (*df*)	*p*
		Global	Male	Female		
Frequency of consumption	Low	881 (96.4)	389 (95.3)	492 (97.2)	1.81 (1)	0.179
	High	33 (3.6)	19 (4.7)	14 (2.8)		
Presence of HED	Absence	840 (91.9)	382 (93.6)	458 (90.5)	2.54 (1)	0.111
	Presence	74 (8.1)	26 (6.4)	48 (9.5)		
Frequency of HED	Low	880 (96.3)	395 (96.8)	485 (95.8)	0.35 (1)	0.555
	High	34 (3.7)	13 (3.2)	21 (4.2)		

*Note. df* = degrees of freedom, HED = heavy episodic drinking.

**Table 4:** Multiple logistic regression models with high-order values, sex, and age as predictors.

Model 1: Frequency of Consumption											
Prediction Accuracy (%):			Global: 83.3			Sensibility: 57.1			Specificity: 84.0		
Predictors	*B*	*SE*	z	*p*	*OR*	*CI_95_*	Likelihood Ratio Test			R^2^ Cox-Snell	R^2^ Nagelkerke
							*χ* ^2^	*df*	*p*		
Age	0.75	0.15	5.04	<0.001	2.12	[1.60; 2.87]	27.17	2	<0.001	0.07	0.25
Sex (male)	0.75	0.40	1.90	0.060	2.12	[0.98; 4.70]					
Conservation	−0.92	0.24	−3.83	<0.001	0.40	[0.24; 0.63]					
Openness to change	1.26	0.34	3.73	<0.001	3.53	[1.88; 7.09]					
**Model 2: Presence of Heavy Episodic Drinking**											
**Prediction Accuracy (%):**			**Global: 72.0**			**Sensibility: 73.9**			**Specificity: 71.8**		
**Predictors**	***B***	***SE***	**z**	***p***	***OR***	***CI_95_***	**Likelihood Ratio Test**			**R^2^** **Cox-Snell**	**R^2^ Nagelkerke**
							***χ*** **^2^**	***df***	***p***		
Age	0.77	0.10	7.76	<0.001	2.16	[1.80; 2.66]	5.32	1	0.021	0.09	0.21
Sex (male)	−0.73	0.27	−2.70	0.007	0.48	[0.28; 0.81]					
Conservation	−0.36	0.16	−2.27	0.023	0.70	[0.51; 0.95]					
**Model 3: Frequency of Heavy Episodic Drinking**											
**Prediction Accuracy (%):**			**Global: 74.20**			**Sensibility: 77.8**			**Specificity: 74.1**		
**Predictors**	***B***	***SE***	**z**	***p***	***OR***	***CI_95_***	**Likelihood Ratio Test**			**R^2^** **Cox-Snell**	**R^2^ Nagelkerke**
							***χ*** **^2^**	***df***	***p***		
Age	0.99	0.17	5.99	<0.001	2.69	[1.99; 3.80]	11.20	2	0.004	0.07	0.25
Sex (male)	−0.42	0.39	−1.06	0.287	0.66	[0.30; 1.40]					
Conservation	−0.53	0.23	−2.33	0.020	0.59	[0.37; 0.91]					
Openness to change	0.82	0.32	2.60	0.009	2.27	[1.26; 4.35]					

**Table 5:** Single logistic regression models with basic values as predictors and controlling for sex and age.

Criterion: Frequency of Consumption											
Value	*B*	*SE*	z	*p*	*OR*	*CI_95_*	Likelihood Ratio Test			R^2^ Cox-Snell	R^2^ Nagelkerke
							*χ* ^2^	*df*	*p*		
Self-direction	0.67	0.26	2.59	0.010	1.95	[1.20; 3.31]	7.61	1	0.006	0.05	0.17
Stimulation	0.45	0.19	2.33	0.020	1.57	[1.09; 2.33]	5.97	1	0.015	0.04	0.16
Hedonism	0.73	0.31	2.31	0.021	2.07	[1.17; 4.03]	6.47	1	0.011	0.04	0.16
Security	−0.39	0.16	−2.45	0.014	0.68	[0.49; 0.92]	6.03	1	0.014	0.04	0.16
Conformity	−0.59	0.17	−3.44	0.001	0.55	[0.39; 0.77]	13.41	1	<0.001	0.05	0.19
Tradition	−0.13	0.18	−0.72	0.472	0.88	[0.62; 1.24]	0.52	1	0.471	0.04	0.14
**Criterion: Presence of Heavy Episodic Drinking**											
Value	***B***	***SE***	**z**	***p***	***OR***	***CI_95_***	**Likelihood Ratio Test**			**R^2^** **Cox-Snell**	**R^2^ Nagelkerke**
							***χ*** **^2^**	***df***	***p***		
Security	−0.27	0.11	−2.39	0.017	0.76	[0.61; 0.95]	5.73	1	0.017	0.09	0.21
Conformity	−0.25	0.11	−2.26	0.024	0.78	[0.63; 0.96]	5.29	1	0.021	0.09	0.21
Tradition	−0.03	0.12	−0.24	0.812	0.97	[0.76; 1.23]	0.06	1	0.812	0.09	0.20
**Criterion: Frequency of Heavy Episodic Drinking**											
**Value**	***B***	***SE***	**z**	***p***	***OR***	***CI_95_***	**Likelihood Ratio Test**			**R^2^** **Cox-Snell**	**R^2^ Nagelkerke**
							***χ*** **^2^**	***df***	***p***		
Self-direction	0.28	0.23	1.22	0.221	1.33	[0.86; 2.14]	1.58	1	0.210	0.06	0.21
Stimulation	0.36	0.19	1.88	0.060	1.43	[1.00; 2.10]	3.84	1	0.050	0.06	0.22
Hedonism	0.67	0.32	2.13	0.033	1.96	[1.10; 3.84]	5.42	1	0.020	0.06	0.23
Security	−0.27	0.16	−1.73	0.084	0.76	[0.56; 1.04]	2.97	1	0.085	0.06	0.22
Conformity	−0.17	0.15	−1.10	0.272	0.85	[0.62; 1.13]	1.23	1	0.268	0.06	0.21
Tradition	−0.24	0.18	−1.33	0.182	0.79	[0.56; 1.12]	1.80	1	0.180	0.06	0.21

*Note.* Only basic values of significant higher-order values are presented: openness to change (self-direction, stimulation, and hedonism) and conservation (security, conformity, and tradition).

## Data Availability

The raw data supporting the conclusions of this article will be made available by the authors on request.

## References

[1] Pan American Health Organization [PAHO] (2020). Regional Status Report on Alcohol and Health in the Americas 2020. https://iris.paho.org/bitstream/handle/10665.2/52705/9789275122211_eng.pdf.

[2] Marshall E.J. (2014). Adolescent Alcohol Use: Risks and Consequences. Alcohol Alcohol..

[3] World Health Organization [WHO] (2018). Global Status Report on Alcohol and Health. https://www.who.int/publications/i/item/9789241565639.

[4] World Health Organization [WHO] (2016). Global Strategy for Women’s, Children’s and Adolescents’ Health (2016–2030). https://platform.who.int/docs/default-source/mca-documents/rmncah/global-strategy/ewec-globalstrategyreport-200915.pdf?Status=Master&sfvrsn=b42b6d22_4.

[5] Brunborg G.S., Halkjelsvik T.B., Moan I.S. (2022). Sports participation and alcohol use revisited: A longitudinal study of Norwegian postmillennial adolescents. J. Adolesc..

[6] Walczak B., Walczak A., Tricas-Sauras S., Kołodziejczyk J. (2023). Does sport participation protect adolescents from alcohol consumption? A scoping review. Int. J. Environ. Res. Public Health.

[7] Perez-Gaido M., Corti J.F., Miscusi A., Schmidt V. (2023). Flow and Personal Accomplishment as Protective Factors of Alcohol Consumption in Young Athletes. Anu. Investig. UBA.

[8] Schmidt V., Celsi I., Molina M.F., Raimundi M.J., García-Arabehety M., Pérez-Gaido M., Iglesias D., González M.A. (2019). Engagement to Sport as a Protective Factor against Alcohol Consumption in Young People. Cuad. Psicol. Deporte.

[9] Schmidt V., Molina M.F., Celsi I., Corti J.F., Raimundi M.J. (2022). Enjoyment and self-realization through sport as protective factors for alcohol consumption: The moderating role of the impulsive sensation-seeking trait. Health Addict..

[10] Castillo I., Molina-García J., Álvarez O. (2010). Importance of Perceived Competition and Motivation to the Mental Health of College Athletes. Salud Pública México.

[11] Ferriz R. (2014). Importance of Satisfaction in Physical Education Classes for Motivation and the Adoption of a Healthy Lifestyle. Ph.D. Dissertation.

[12] Roccas S., Sagiv L. (2010). Values and Behavior. Taking a Cross-Cultural Perspective.

[13] Adell F.L., Castillo I., Alvarez O. (2019). Personal and sport values, goal orientations, and moral attitudes in youth basketball. J. Sport. Psychol..

[14] Corti J.F., Raimundi M.J., Celsi I., Alvarez O., Castillo I. (2023). The moderating effect of athletes’ personal values on the relationship between coaches’ Leadership behaviors and the personal and social skills of young basketball players. Sustainability.

[15] Doran C.J. (2009). The role of personal values in fair trade consumption. J. Bus. Ethics.

[16] Schwartz S.H., Zanna M.P. (1992). Universal in the content and structure of values: Theoretical advances and empirical tests in 20 countries. Advances in Experimental Social Psychology.

[17] Schwartz S.H., Cieciuch J., Vecchione M., Davidov E., Fischer R., Beierlein C., Ramos A., Verkasalo M., Lönnqvist J.E., Demirutku K. (2012). Refining the theory of basic individual values. J. Pers. Soc. Psychol..

[18] Castillo I., Adell F.L., Alvarez O. (2018). Relationships between personal values and leadership behaviors in basketball coaches. Front. Psychol..

[19] Bardi A., Schwartz S.H. (2003). Values and behaviour: Strength and structure of relations. Pers. Soc. Psychol. Bull..

[20] Balaguer I., Castillo I., Quested E., Duda J.L., Whitehead J., Telfer H., Lambert J. (2013). How do values relate to motivation?. Values in Youth Sport and Physical Education.

[21] Ring C., Whitehead J., Gürpınar B., Kavussanu M. (2023). Sport values, personal values and antisocial behavior in sport. Asian J. Sport Exerc. Psychol..

[22] Arriaga Martínez J.L. (2019). Personal Values, Alcohol Advertising on Social Media and Adolescent Alcohol Consumption. Ph.D. Dissertation.

[23] Rodríguez J.A.G.D.C., López-Sánchez C., Soler M.C.Q., Del Castillo-López Á.G., Pertusa M.G., Campos J.C.M., Inglés C.J. (2013). Predictive models of alcohol use based on attitudes and individual values. J. Drug Educ..

[24] Nordfjærn T., Brunborg G.S. (2015). Associations between Human Values and Alcohol Consumption among Norwegians in the Second Half of Life. Subst. Use Misuse.

[25] Danioni F., Villani D., Ranieri S. (2023). Personal values and substance use in adolescence and young adulthood: Risk or protective factors?. Subst. Use Misuse.

[26] Secretariat of Comprehensive Policies on Drugs [SEDRONAR] (2017). National Study in Population Aged 12 to 65 Years, on Psychoactive Substance Consumption. Argentina 2017. https://www.observatorio.gov.ar/media/k2/attachments/15.pdf.

[27] Argentine Ministry of Health [AMH] (2018). Global School-Based Student Health Survey. https://bancos.salud.gob.ar/sites/default/files/2020-01/encuesta-mundial-salud-escolar-2018.pdf.

[28] Argentine Ministry of Health [AMH] (2019). Diagnosis of the Alcohol Consumption Situation in Argentina and Recommendations for the Implementation of Health Policies. https://bancos.salud.gob.ar/sites/default/files/2020-01/consumo_alcohol_argentina-11-2019.pdf.

[29] Griffin K.W., Lowe S.R., Botvin C., Acevedo B.P. (2019). Patterns of adolescent tobacco and alcohol use as predictors of illicit and prescription drug abuse in minority young adults. J. Prev. Interv. Community.

[30] National Institute of Statistics and Census [INDEC] (2023). National Survey of Consumption and Care Practices 2022. https://www.indec.gob.ar/ftp/cuadros/sociedad/encoprac_2022.pdf.

[31] Schwartz S.H. (2021). A repository of Schwartz Value Scales with instructions and an introduction. Online Read. Psychol. Cult..

[32] Beramendi M., Zubieta E. (2017). Validation of the 40 and 21 items versions of the Portrait Values Questionnaire in Argentina. Psychologia.

[33] Castro Solano A., Nader M. (2006). The Assessment of Human Values with Schwartz’s Portrait Values Questionnaire. Interdisciplinaria.

[34] Fernandez Liporace M., Ongarato P., Saavedra E., Casullo M.M. (2005). Values in Adolescent Students: An Adaptation of Schwartz’s Portrait Values Questionnaire. Rev. Iberoam. Diagnóstico Y Evaluación-E Avaliação Psicológica.

[35] Schwartz S.H. (2003). A proposal for measuring value orientations across nations. Quest. Dev. Package Eur. Soc. Surv..

[36] Imhoff D., Brussino S. (2013). Exploratory study of the psychometric characteristics of the portrait values questionnaire in the context of Córdoba-Argentina. Rev. Colomb. Psicol..

[37] Cremonte M., Ledesma R.D., Cherpitel C.J., Borges G. (2010). Psychometric properties of alcohol screening tests in the emergency department in Argentina, Mexico and the United States. Addict. Behav..

[38] Bush K., Kivlahan D.R., McDonell M.B., Fihn S.D., Bradley K.A. (1998). The AUDIT alcohol consumption questions (AUDIT-C): An effective screening test for problem drinking. Ambulatory Care Quality Improvement Project (ACQUIP). Alcohol Use Disorders Identification Test. Arch. Intern. Med..

[39] Conde K., Gimenez P., Cremonte M. (2018). Characteristics of screening instruments for alcohol use disorder in young university students in Argentina. Rev. Clínica Y Salud.

[40] Borg I., Groenen P.J., Mair P. (2018). Applied Multidimensional Scaling and Unfolding.

[41] Bartholomew D.J., Steele F., Moustaki I., Galbraith J.I. (2008). Analysis of Multivariate Social Science Data.

[42] Mair P., Borg I., Rusch T. (2016). Goodness-of-fit assessment in multidimensional scaling and unfolding. Multivar. Behav. Res..

[43] Cieciuch J., Schwartz S.H. (2012). The number of distinct basic values and their structure assessed by PVQ-40. J. Pers. Assess..

[44] Arias B., Verdugo M.A., Badía M., Arias B., Crespo M. (2008). Development of a confirmatory factor analysis example with LISREL, AMOS and SAS. Metodología en la Investigación Sobre Discapacidad: Introducción al Uso de las Ecuaciones Estructurales.

[45] Gana K., Broc G. (2018). Structural Equation Modeling with Lavaan.

[46] R Core Team (2021). R: A Language and Environment for Statistical Computing.

[47] Schwartz S.H. (2012). An overview of the Schwartz theory of basic values. Online Read. Psychol. Cult..

[48] Conde K., Brandariz R.A., Lichtenberger A., Cremonte M. (2018). The effectiveness of a brief intervention for reducing adolescent alcohol consumption. Rev. Cienc. Salud.

[49] Rial Boubeta A., Golpe Ferreiro S., Araujo Gallego M., Braña Tobio T., Varela Mallou J. (2017). Validation of the Alcohol Use Disorders Identification Test (AUDIT) in the Spanish adolescent population. Psicol. Conduct..

[50] Coelho G.L.H., Hanel P.H.P., Vilar R., Monteiro R.P., Gouveia V.V., Maio G.R. (2018). Need for Affect and Attitudes toward Drugs: The Mediating Role of Values. Subst. Use Misuse.

[51] Schmidt V., Martucci F., Di Puglia G., Lo Giusto O., Rijana I., Alvarez Iturain A. (2019). Qualitative study on risk and care practices in nightlife scenarios of massive presence in Buenos Aires. Salud Colect..

[52] Magidson J.F., Robustelli B.L., Seitz-Brown C.J., Whisman M.A. (2017). Activity enjoyment, not frequency, is associated with alcohol-related problems and heavy episodic drinking. Psychol. Addict. Behav..

[53] Monteagudo M.J., Ahedo R., Ponce de León A. (2017). The benefits of youth leisure and its contribution to human development. OBETS Rev. Cienc. Soc..

[54] Márquez A.T., Alonso-Castillo M.M., Gómez Meza M.V., Alonso Castillo B.A., Rodríguez N.N.O., Armendáriz-García N.A. (2016). Motivations for sports physical activity and alcohol consumption in high school students. Cienc. Y Enfermería.

